# Delivering Phage Products to Combat Antibiotic Resistance in Developing Countries: Lessons Learned from the HIV/AIDS Epidemic in Africa

**DOI:** 10.3390/v10070345

**Published:** 2018-06-27

**Authors:** Tobi E. Nagel

**Affiliations:** Phages for Global Health, 383 62nd Street, Oakland, CA 94618, USA; tobi@phagesforglobalhealth.org; Tel.: +1 650-888-5522

**Keywords:** bacteriophage, phage therapy, antimicrobial resistance, antibiotic, global health, developing countries, infectious disease

## Abstract

The antimicrobial resistance (AMR) crisis and HIV/AIDS epidemic exhibit many parallels. In both, infectious diseases have caused millions of deaths worldwide, with AMR expected to kill even more people each year than HIV/AIDS did at its peak. In addition, both have required or will require new classes of drugs for control. For HIV/AIDS, development of vital antiretroviral drugs (ARVs) was accomplished in several stages: expanding public awareness about the disease, gathering commitment from the international community to tackle the problem, and eventually establishing policies and global funds to deliver new therapeutics. For AMR, the pursuit of new antimicrobials appears to be following a similar trajectory. This paper examines how lessons and processes leading to ARVs might be applied to developing AMR drugs, in particular bacteriophages (phages). These possess many essential characteristics: inexpensive manufacture, rapid drug development, and a ready means to prevent phage-resistant microbes from emerging. However, the broad application of phage-based products has yet to be fully demonstrated, and will require both international coordination and modified regulatory policies.

## 1. Introduction

**Figure 1:**
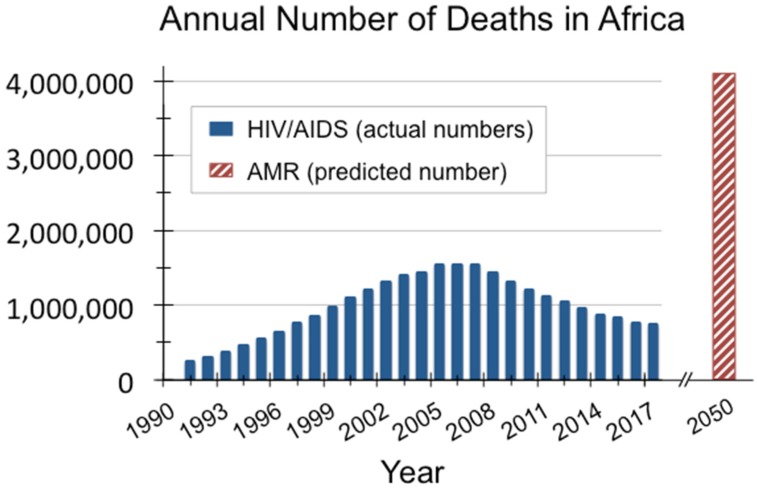
Africa was hardest hit by the HIV/AIDS crisis, and is expected to suffer the highest mortality per capita from AMR by 2050 [1,2].

HIV/AIDS spurred the deadliest epidemic in modern history. At its peak, the disease caused 1.9 million deaths per year worldwide [1]. The global antimicrobial resistance (AMR) crisis will be even bigger: by 2050, antibiotic-resistant infections are predicted to kill roughly 10 million people annually [2]. As with HIV/AIDS, the developing world will be hardest hit by AMR, with nearly 90% of expected deaths occurring in those countries. For example, in Africa alone, the annual loss of life from AMR is forecasted to be 4.15 million, surpassing the 1.54 million deaths caused by HIV/AIDS in Africa during 2005, the worst year of that crisis (see Figure 1).

Notably, when the HIV/AIDS epidemic began, there were no effective drugs available to treat the emerging infectious disease. Similarly, there are now no conventional treatment options in the face of antimicrobial resistance. Just as new drug classes—namely antiretrovirals (ARVs)—were needed to mitigate HIV/AIDS, new antimicrobial drugs will be required to tackle AMR.

Phages—viruses that can kill both antibiotic-sensitive and antibiotic-resistant bacteria—are a key drug class that could save many lives threatened by the AMR crisis. Phages have been used as antibacterial agents for nearly 100 years in the former Soviet Union, and they are now undergoing a renaissance in other countries due to the growing AMR problem [3,4,5,6,7]. In addition to being able to provide therapeutic options when no others exist, phages are inherently inexpensive to isolate, have relatively short product development time frames, and no major reported side effects, despite their decades of use. Unlike traditional antibiotics, phage products can readily be designed to thwart development of resistance. Nonetheless, phage therapy has yet to be fully proven and implemented in most regions of the world.

Substantial funding and appropriate regulatory structures would be needed to develop and deploy phage products to the vast number of people who will be impacted, as was necessary for ARVs. This publication examines what lessons from the HIV/AIDS epidemic might be applied to phages and the AMR crisis; specifically, what funding programs and regulatory modifications enabled rapid research and development of a new class of drugs, followed by large-scale manufacturing and distribution of those drugs to developing countries.

## 2. Support for ARV Development and Delivery

The worldwide response to the HIV/AIDS crisis was unprecedented in terms of the speed with which ARVs were developed and applied clinically, especially in low and middle income countries (LMIC). These drugs ultimately transformed HIV/AIDS from a disease whose sufferers had a life expectancy of approximately one year to our current situation, in which HIV-positive individuals can experience a nearly normal life span [8]. An estimated 11 million lives have now been saved, and almost 2 million babies have been born HIV-free [9]. However, because of limitations in infrastructure and delivery capacity in most LMIC, many people globally have yet to fully benefit from ARVs.

The first AIDS case was diagnosed in 1981, and the first ARV to treat HIV/AIDS, azidothymidine (AZT), was approved in 1987, with much of the initial funding coming from the US government [10]. Once the huge scale of the HIV/AIDS epidemic became apparent, market incentives then motivated private investment into additional ARV drug development, particularly in the US. Over time, combinations of three different ARVs proved much more effective than AZT alone, with little or no resistance developing against the drugs, and these so-called highly active antiretroviral therapy (HAART) combinations became the standard of care. As will be discussed below, combinations of phages will undoubtedly also be required to stave off future bacterial resistance to phage products.

ARVs were eventually deployed to the developing world. The global community went through several stages of grappling with the problem before achieving some success: first comprehending the massive scale of the disease and disseminating that information; then mobilizing global resolve to address the issue; and eventually, establishing substantial international funding sources, particularly to deliver ARVs to LMIC [11]. Millions of deaths potentially could be prevented if a similar distribution of effective antibacterial agents were expedited. Let us first review how drug development and distribution was accomplished for the HIV/AIDS crisis, then consider how this could inform the global response to AMR.

### 2.1. Understanding the Scope of the Crisis

As early as 1983, the World Health Organization (WHO) convened its first meeting on HIV/AIDS and began formal international surveillance of the disease [11]. Two years later, the WHO and the US Department of Health and Social Services hosted the first International AIDS Conference, and in 1987, the WHO initiated the Global Program on AIDS, with a primary goal of raising awareness about the growing epidemic. That same year, the US Center for Disease Control and Prevention begin a large-scale public service campaign. This was followed by the first World AIDS Day in 1988, and the launching of the Red Ribbon Project in 1991, from which came the popular international symbol for AIDS. These and other awareness-raising efforts were eventually followed by formal resolutions from national and international organizations determined to combat the problem.

### 2.2. Generating Worldwide Commitment

New governmental organizations and processes were set up to coordinate policy development and global activities to tackle HIV/AIDS. Most notable of these was the Joint United Nations program on HIV/AIDS (UNAIDS), which began operations in 1996 [12]. In 2001 the UN General Assembly held a Special Session on AIDS—the first time that global body had ever focused a full session on a single disease [10]. A key outcome of the meeting was the formal Declaration of Commitment on HIV/AIDS, which included a call to establish an international global fund. Less than two years later, the WHO introduced the “3 by 5” initiative, a program so-named because it aimed to treat 3 million people in developing countries with ARVs by 2005 [13]. Importantly, international leaders also began to recognize the HIV/AIDS epidemic as both a health crisis and a global security issue, with formal statements issued by heads of state as well as by the UN Security Council [11]. With global will more fully engaged, steps were taken to find the monetary resources to achieve the specified goals.

### 2.3. Establishing Funding Sources for Delivering Medicines

Financing for the HIV/AIDS crisis came in stages of increasingly larger investments. The World Bank Multicountry AIDS Program (MAP), established in 2000, was the first international program, with disbursements totaling $500 million in the initial funding round [14]. After two or more years of international deliberations, the Global Fund to Fight AIDS, Tuberculosis, and Malaria (GFATM) was formalized in 2002 [15]. Instituted as a public-private partnership, the GFATM initially called for $1 billion, but received $1.9 billion in pledges by the time it became operational. Funding came from private individuals (typically in the order of hundreds of thousands of dollars), private corporations (the first being a $1 million donation), and private foundations and governments (with contributions ranging up to $200 million each). While this was a substantial leap forward in funding, it was still notably less than the $7–10 billion yearly investment that Kofi Annan, then UN Secretary-General, targeted as necessary to address the epidemic, especially in LMIC [16]. In 2003, the most significant funding materialized with the launch of the US President’s Emergency Plan For AIDS Relief (PEPFAR), which allocated $15 billion for the first 5 years, then expanded to $48 billion in 2008 [17]. To date PEPFAR remains the largest public health investment program from a single country.

### 2.4. Inventing Mechanisms to Decrease Drug Prices in Developing Countries

In addition to gathering the financial resources needed, several regulatory modifications were enacted to facilitate faster and therefore more cost-effective delivery of ARVs to the developing world. In 1997, the US Congress passed the FDA Modernization Act in order to accelerate the drug approval process and loosen restrictions on communications regarding off-label use for potential HIV drugs [18]. And in 2004, the FDA issued new guidance policies to expedite approval of co-packaged and combination therapies aimed at developing countries [11]. These regulatory adaptations would ultimately prove to be significant in facilitating delivery of the drugs to the populations that needed them. Reforms in regulatory structures likely will be necessary for new AMR drugs as well, particularly for phage-based products.

New financial and legal resolutions were also put in place to help reduce the costs of ARVs. In 2000, the UNAIDS and WHO negotiated the Accelerating Access Initiative, an agreement with five major pharmaceutical companies to provide HIV/AIDS drugs to developing countries at decreased prices [10]. In addition, in 2001 the World Trade Organization established the Doha Declaration, which allowed developing countries access to generic drugs for public health crises, even without formal patent approval in each country [19]. Some companies also joined the Medicines Patent Pool, which facilitates licensing for manufacturing of generics for LMIC, speeding up the access to drugs in those countries [20]. Similar programs will undoubtedly facilitate the delivery of new AMR therapeutics to LMIC.

While these various support mechanisms for LMIC were initially funded by industrialized countries, developing countries themselves eventually began to underwrite the local delivery of ARVs. By 2013, roughly half of all costs for HIV treatment in sub-Saharan Africa were covered by in-country sources [21]. And in a few countries, particularly Angola, Botswana, and South Africa, more than 80% of the financing came from domestic funds. Some countries, such as Cape Verde and Cote d’Ivoire, are also utilizing creative funding schemes such as taxes on tobacco and alcohol to raise money for HIV/AIDS treatment. However, there is still less domestic funding across Western and Central Africa (15–29%).

## 3. Current Support for AMR Drug Development

As the world has been coming to grips with AMR, we have begun to progress through similar stages as with the HIV/AIDS epidemic. However, as discussed below, we are still at a relatively early point in this overall process. In order to effectively fund new drug development for AMR, including for phages, the global community will need to first more fully comprehend the scale of the crisis, which will then motivate international commitment to combat the problem, finally leading to the establishment of the financial resources and regulatory modifications needed.

### 3.1. Understanding the Scope of the Crisis

Resistant strains to the first small molecule antibiotic, penicillin, were identified even before penicillin was introduced in 1943 [22]. As new antibiotics have become available, resistance was recorded as early as one year after the first clinical use. This reality must be addressed as new antibacterials are developed for AMR.

Over the past decades, the scientific and public health communities have been documenting the growing rates of antibiotic resistance in specific bacterial strains and geographic regions. The WHO’s first report on AMR, published in 2014, summarized the global resistance patterns for seven bacteria of major concern: *Escherichia coli*, *Klebsiella pneumoniae*, *Staphylococcus aureus*, *Streptococcus pneumoniae*, *Nontyphoidal Salmonella*, *Shigella*, and *Neisseria gonorrhoea* [23]. Strikingly, resistance to carbapenems, considered the last-resort antibiotics, was documented in the majority of the reporting countries, including some resistance rates up to 54%. The report highlighted that a post-antibiotic era is “far from being an apocalyptic fantasy, [but] is instead a very real possibility for the 21st century”.

By July 2014, this data and others had prompted the UK Prime Minister to commission a study analyzing the key components of the crisis and proposing tangible steps that the international community could take to surmount it. Funded in partnership with the Wellcome Trust, the predictions from the resulting *Review on Antimicrobial Resistance* were astounding: by 2050, AMR is expected to cause over 10 million deaths each year and cost the global economy a total of $100 trillion [2,24]. In addition, a 2016 report from the World Bank Group estimates that AMR could push 28.3 million people into extreme poverty [25].

These reports served as wake-up calls for many, and the authors of the *Review on Antimicrobial Resistance* emphasized that there is still a primary need for AMR awareness-raising campaigns globally, despite the fact that numerous organizations have been spreading knowledge about AMR for decades. One of the earliest was the Alliance for the Prudent Use of Antibiotics, founded in 1981 by Dr. Stuart Levy, a leading researcher on molecular antibiotic efflux mechanisms [26]. In 2001 the European Union Council issued a formal recommendation on the judicious use of antimicrobial agents in human medicine [27]. This eventually led to the inauguration of European Antibiotic Awareness Day in 2008, now held yearly [28]. More recently, the WHO conducted a multi-country public awareness survey on AMR during the fall of 2015 to understand how best to deliver information and what topics to focus on [29]. Two months later, the first World Antibiotic Awareness Week was launched [30].

### 3.2. Generating Worldwide Commitment

Calls to action have come from various authoritative sources. In 2004 and 2008, the Infectious Diseases Society of America highlighted key causes and dangers of AMR, and proposed specific ways to stimulate investment in antibiotic research and development [31,32]. And in 2013, a panel of global experts published the Lancet Infectious Diseases Commission, a summary document on antibiotic resistance which called for coordinated international efforts to contain AMR, emphasizing that individual countries cannot effectively address the issue on their own [4]. A month after the WHO 2014 report on AMR, the Director of the Wellcome Trust, Dr. Jeremy Farrar, and Professor Mark Woolhouse of the University of Edinburgh published a Comment in *Nature* calling for the establishment of an intergovernmental panel on antimicrobial resistance [33].

To date, a global panel for AMR has not been formed. An oversight body of this type could more effectively coordinate international efforts, analogous to the role of UNAIDS for HIV/AIDS. In the meantime, worldwide forums have issued formal declarations on AMR, and both international and national bodies have created action plans. In May 2015, the World Health Assembly approved the Global Action Plan for Antimicrobial Resistance, which enumerates key strategies, including the need to increase AMR awareness globally and to develop policies for attracting more investment into new medical interventions [34]. It also called upon all Member States to establish national action plans for AMR by 2017. Thus far, 57 countries have formalized such plans [35]. Another major step forward was the 2016 meeting of the UN General Assembly focused on AMR, with plenary panel discussions on the need for multisectoral solutions to the problem [36]. Additionally, in July 2017, the G20 called for the creation of a Global R&D Collaboration Hub on AMR that would coordinate international funding efforts—the first G20 Declaration that has included R&D for public health [37]. The search for the appropriate individual to lead that hub began in early 2018.

### 3.3. Establishing Funding Sources for Delivering Medicines

In order to stall HIV/AIDS to the current level, PEPFAR, the largest funder for this disease, has spent over $70 billion since 2004 [38]. By comparison, the UK-commissioned *Review on Antimicrobial Resistance* estimated that $40 billion will be needed over 10 years to adequately address the global AMR problem. This represents only about 0.05% of the total amount that G20 countries currently spend on healthcare, and it is quite small compared to the projected $100 trillion that will be needed if AMR is not addressed. Of the total $40 billion proposed for AMR, likely $16 billion would be necessary to boost a new antibiotic pipeline, assuming a traditional drug development process and associated costs. However, phage manufacturing could be less expensive, and thus, do more with less.

As an incremental step toward gathering the financial support, the *Review on Antimicrobial Resistance* called for establishment of a Global Innovation Fund for AMR, with seed funding of $2 billion for 5 years. The UK and China responded by initiating such a fund in 2015, with each country pledging £50 million (equivalent to roughly $66 million); the Bill & Melinda Gates Foundation also agreed to contribute. While this falls far short of the recommended amount, it is a start. Recall that the analogous HIV funding began with $500 million before eventually growing to over $70 billion.

In early 2017, the Global Union for Antibiotics Research and Development (GUARD) published its own set of recommendations on specific steps for addressing AMR [39]. Its proposal included three separate funding mechanisms to stimulate a pipeline of antibiotics:

(1) Global Research Fund to build up infrastructure and increase the number of scientists working in the AMR field ($200 million/year for 10 years)
(2) Global Development Fund to provide forgivable loans primarily to small and medium-sized enterprises with the goal of pushing ten “high-need” products to market over a decade ($200 million/year for 10 years)
(3) Global Launch Reward of $1 billion for successfully delivering a commercial product that meets pre-specified AMR therapeutic goals

Both the *Review on Antimicrobial Resistance* and the GUARD report recommended a balance of so-called “pull” vs. “push” financial incentives. Push incentives, such as grants and forgivable loans, would help move the initial stages of R&D forward. Pull incentives, typified by the Global Launch Reward, would motivate companies to progress through the final, more costly stages of drug development by providing a monetary award that essentially lowers the financial risk. Economic experts generally agree that numerous push incentives are currently available, but pull incentives are sparse.

Other large AMR financing mechanisms have been established in Europe and the US. NewDrugs4BadBugs (ND4BB) was created in 2012 by the European public–private partnership Innovative Medicines Initiative (IMI). The goal of ND4BB is to bring together academic, industry and biotech groups to find new ways to overcome the practical challenges of developing new antimicrobial drugs [40]. To date, this program includes eight projects totaling EUR 650 million (approximately $758 million), with roughly half of the initial funding coming from IMI and the remainder from large pharmaceutical companies.

In the US, much of the funding for AMR comes through the Biomedical Advanced Research and Development Authority (BARDA), which was established in 2010 to address a number of public health emergencies. BARDA works through public-private partnerships with pharmaceutical and biotech companies, providing non-dilutive funding to help companies develop new antimicrobials. In 2016, BARDA partnered with the Wellcome Trust and the AMR Centre in the UK, as well as with the US NIAID, to launch a global business accelerator program called the Combating Antibiotic-Resistant Bacteria Biopharmaceutical Accelerator (CARB-X) [41]. To date, CARB-X has raised $500 million and funded 33 companies from 7 countries. Since 2004, the Wellcome Trust has also invested approximately $400 million in drug-resistant infection activities, and the US NIAID/NIH has contributed roughly $340 million per year since 2013 [42,43].

Another financial instrument, InnovFin Infectious Diseases, was founded in 2015 under the European Investment Bank, with estimates suggesting that it may provide up to $350 million [39]. And in 2016, the Global Antibiotic Research & Development Partnership (GARDP) was established by the WHO and the non-profit organization Drugs for Neglected Diseases initiative (DNDi) [44]. This program builds on DNDi’s experience in developing drugs for neglected diseases that particularly impact LMIC, and it will include both push and pull financial incentives. The GARDP 2017–2023 plan includes raising the equivalent of $315 million and delivering four new therapeutics. While still shy of the projected $40 billion that will be needed, these and other smaller funds are important steps toward financing new therapeutics to overcome AMR.

### 3.4. Inventing Mechanisms to Decrease Drug Prices in Developing Countries

Several other strategies have been launched or proposed to foster new drug development. In 2012, the Generating Antibiotic Incentives Now (GAIN) legal provision was enacted in the US to incentivize pharmaceutical companies [45]. This law extended the exclusivity period by five years for specified antibiotic categories (those that target particularly concerning pathogens), thus prolonging the time that such drugs can be sold without competition from generics. In addition, the authors of the *Review on Antimicrobial Resistance* have proposed a tax on pharmaceutical companies, with the collected levies used to fund pull incentive awards for successful commercialization of new antimicrobials [24]. The rationale is that since antibiotics are such an integral component of modern medical systems, pharmaceutical companies which sell drugs other than antibiotics—such as those for oncological or surgical applications—indirectly benefit from having effective antibiotics available. The proposed tax would also directly incentivize drug companies to keep the antibiotic pipeline robust: companies that did invest in AMR drug development could receive tax credits and would be eligible for the market entry awards if their product delivery were successful. Another potential pull incentive is advance market commitments (AMCs), wherein drug developers would be guaranteed a set price and volume of sales for production of drugs that target specific pathogens of concern. In recent years, AMCs have been utilized to motivate development of a pneumonia vaccine [46].

Given that the first significant finances for AMR have only been committed in the past few years, the global community is still in the early stages of fundraising. Realistically, most of the money for LMIC will need to come from industrialized countries, as was the case for HIV/AIDS; developing countries simply do not have the resources. Of course, AMR truly is a global problem, since bacteria readily move across borders.

Nonetheless, the fundraising goals appear achievable, as evidenced by the worldwide response to HIV/AIDS. Now is the key time to strategize about how to use funds most efficiently. While conventional development of new antibiotics will certainly be important, that approach is typically an expensive and lengthy one. In addition, bacteria will undoubtedly continue to evolve resistance mechanisms to new classes of drugs. An ideal solution would be to invest in low-cost drugs that reduce development of resistance.

## 4. Attributes of Phage-Based Products

Phage-based drugs already provide such an option in the Eastern European countries of Georgia, Poland, and Russia [3,5,7]. Let us now discuss some of the currently available phages, and the general characteristics of those types of products that could be particularly beneficial for addressing the immense AMR crisis in the developing world, especially in the context of the funding environment described above.

### 4.1. Inexpensive Drugs for Infectious Diseases

Unlike ARVs for HIV/AIDS, which were very expensive drugs to develop, phages are inherently inexpensive to isolate. Indeed, therapeutic phages were first developed for patients in the 1920s using the relatively simple laboratory equipment that was available at that time [5,7]. In the former Soviet Union, phages have been utilized clinically for about 100 years. These include products targeting bacteria that underlie diarrhea, wound infections, and urinary tract infections, amongst others, including both antibiotic-sensitive and antibiotic-resistant strains. Today, phages can be bought over-the-counter in Georgia and Russia for as little as the equivalent of $1–2 per dose, though a full course of personalized phage treatment may cost as much as $1000–3000 per patient depending on the dose level and treatment duration [47,48,49,50].

Phages in Georgia, Poland, and Russia are manufactured under conditions that do not meet formal Western cGMP (Current Good Manufacturing Practice) requirements, but that do adhere to a different set of strict quality control standards—a key reason why phages are available in those countries at relatively low prices. These products have been used for decades with no reported major adverse events. Another non-cGMP regulatory system called *magistral preparations* was approved for phages in Belgium in early 2018 [51]. Under this system, phages are considered active pharmaceutical ingredients if they are produced as per a formally approved monograph, then quality tested in batches at a Belgian Approved Laboratory, accredited by the national regulatory authority. Both the Eastern European and Belgian manufacturing systems provide safe phage products at much lower costs than if they were prepared under conventional cGMP systems. Similar modifications to existing international manufacturing requirements would facilitate production of phages for AMR, just as the FDA adjusted policies to expedite regulatory approval for HIV/AIDS drugs. Given the huge quantities of antibacterial agents that will be needed to overcome antibiotic-resistant infections globally, this could be essential for providing quality products at reasonable costs.

### 4.2. Short Product Development Time Frames

Another key attribute of phages is that these products can be developed in very short time frames, which can both help to keep costs down and enable rapid responses to infection outbreaks. As an example, in 2016, a patient in San Diego, California, received emergency phage treatment after contracting a multi-drug resistant strain of *Acinetobacter baumannii* [52]. The search for appropriate phages for the patient began on February 21, and the first phage treatment was administered on March 15—just 23 days later [53]. During those few short weeks, previously isolated and characterized phages from several groups in the US and Europe were tested against bacteria isolated from the patient, then the selected phages went through two rounds of purification to ensure low endotoxin levels. In that same time period, the team managing the case submitted an Emergency Investigational New Drug application to the FDA and were given official approval to deliver the phages to the patient. After multiple phage doses were administered, the patient’s infection completely cleared. This dramatic case highlights how rapidly an international community of scientific and clinical experts can address an acute infectious disease problem with phages. Also, while the quality of these phages was rigorously monitored by the FDA, they were not manufactured according to cGMP.

### 4.3. Decreased Probability of Resistance Development

Several tactics have been utilized to minimize the possibility that bacteria will eventually develop resistance to phages. The most common are mixing phages that target different bacterial epitopes and rotating the phage combinations at regular intervals [54]. Recall that combinations of ARVs were also required to address HIV/AIDS, and the FDA enacted guidelines for expediting the approval of such combinations, specifically with developing countries in mind [11]. Thus, there is precedence for revising standard regulatory processes to facilitate delivery of essential drug combinations.

Phage treatments are most effectively made of routinely shifting, carefully selected mixtures (termed “cocktails”) that target multiple bacterial epitopes proliferating in a given locality [53]. Thus, what makes phages good at targeting antibiotic resistance is also what makes it impractical to manufacture them under a cGMP system—which works best for fixed chemical or biological matter, against which AMR can develop rapidly.

It is critically important to select appropriate phages for each cocktail. This includes avoiding phages that can transduce virulence genes through their local environment (e.g., lysogenic phages), as well as excluding phages that contain toxin genes which potentially could be transferred to the bacterial hosts. Regulatory systems must address these possible risks by rigorously controlling for them.

An ideal situation might be to have banks of pre-approved phages in each country or region. Centralized laboratories could regularly test those phages against bacterial populations currently circulating in each country, modifying the selected phage combinations as needed, and routinely adding new phages to the banks through certified approval processes. Local institutions and universities could potentially contribute phages to the banks, thereby providing incentives and opportunities for in-country development. Such a system would both address the need for altering phage mixtures to avoid resistance development, and also enable rapid responses to sudden disease outbreaks.

This is essentially the arrangement that has been in place the former Soviet Union for decades, and it has proven to be effective. It is notable that Georgia—which is officially categorized by the World Bank as an LMIC—is already showing how this system of delivering reasonably-priced, routinely-updated phage products can be accomplished in a developing country.

## 5. Final Thoughts

AMR knows no national boundaries, so overcoming it will take coordinated efforts from countries worldwide working together, likely overseen by a centralized international panel. Innovations in science, financing mechanisms, and regulatory processes will all be required to expedite the path forward. In this context, phage-based products could be an important part of the solution, since they can potentially provide effective and affordable options for killing antibiotic-resistant bacteria, with reduced probability of resistance development.

However, if the global response to AMR follows a similar pattern as that for HIV/AIDS, the worldwide community must focus more on raising awareness and motivating national and international commitment to the problem before sufficient funds will become available. Realistically, many other factors must also be in place to overcome AMR, such as improvements in water sanitation, appropriate use of existing antibiotics, suitable low-cost diagnostics, adequate surveillance, and effective local health systems, amongst others. It is an overwhelming, complex crisis, but the achievements that have been realized with the HIV/AIDS epidemic demonstrate that the global community is capable of bringing together the resources and creative solutions to surmount a problem as big as AMR.
